# Implementation, context and complexity

**DOI:** 10.1186/s13012-016-0506-3

**Published:** 2016-10-19

**Authors:** Carl R. May, Mark Johnson, Tracy Finch

**Affiliations:** 1 Faculty of Health Sciences, University of Southampton, Building 67 (Nightingale), University Road, Highfield, Southampton, SO17 1BJ UK; 2 University Hospital Southampton NHS Foundation Trust, Southampton, UK; 3 NIHR CLAHRC Wessex, University of Southampton, Southampton, UK; 4 NIHR Southampton Biomedical Research Centre, University Hospital Southampton NHS Foundation Trust and University of Southampton, Southampton, UK; 5 Institute of Health and Society, Newcastle University, Newcastle upon Tyne, UK

## Abstract

**Background:**

Context is a problem in research on health behaviour change, knowledge translation, practice implementation and health improvement. This is because many intervention and evaluation designs seek to eliminate contextual confounders, when these represent the normal conditions into which interventions must be integrated if they are to be workable in practice.

**Discussion:**

We present an ecological model of the ways that participants in implementation and health improvement processes interact with contexts. The paper addresses the problem of context as it affects processes of implementation, scaling up and diffusion of interventions. We extend our earlier work to develop Normalisation Process Theory and show how these processes involve interactions between mechanisms of resource mobilisation, collective action and negotiations with context. These mechanisms are adaptive. They contribute to self-organisation in complex adaptive systems.

**Conclusion:**

Implementation includes the translational efforts that take healthcare interventions beyond the closed systems of evaluation studies into the open systems of ‘real world’ contexts. The outcome of these processes depends on interactions and negotiations between their participants and contexts. In these negotiations, the plasticity of intervention components, the degree of participants’ discretion over resource mobilisation and actors’ contributions, and the elasticity of contexts, all play important parts. Understanding these processes in terms of feedback loops, adaptive mechanisms and the practical compromises that stem from them enables us to see the mechanisms specified by NPT as core elements of self-organisation in complex systems.

## Background

Context is a problem for implementation science. Researchers in the field have sought to identify, characterise and explain the mechanisms implicated in individual behaviour change and collective action for many years. They have sought to develop theoretical models and empirical research instruments, as well as practical toolkits that foster the implementation of innovations in practice. Against this background, context is an important *practical* problem for complex intervention and implementation trials. These are driven by a model of research that aims to show the operation of causal mechanisms, eliminate confounders and measure outcomes. The conceptual division between an intervention and its environment that is an inevitable consequence of trial design means that contexts are often framed as sources of obduracy and interference with the smooth delivery of the trial.

Attempts to understand the dynamics of implementation and to evaluate their effects are either front-loaded into clinical trials in the form of programme theories that specify their expected mode of operation and outcomes or revealed in retrospective ‘process’ evaluations of activity over time and the effects of this activity on outcomes [1, 2]. This means that implementation theory and empirical research are skewed in favour of the beginnings of implementation-integration journeys. Longitudinal studies that specifically investigate sustainability and scaling up, and that focus on implementation processes, are rare. We thus know rather less than we should about the mechanisms involved in adaptation and sustainability over time.

There is, however, a substantial literature on aspects of context, relevant to implementation research. It draws attention to factors that play out as ‘barriers and facilitators’ to specific interventions ranging from improving hand-washing in hospital [3]; pain management [4]; shared decision-making about treatment [5] and management of the end of life [6]. The problem for implementation science, however, is that contextual ‘confounders’ that act in this way in fact constitute the normal conditions of practice and are rarely taken into account in that form. As reviews by Lau et al. [7, 8] have shown, changes in policy direction, organisational turbulence and the exigencies of everyday work in complex healthcare settings, all affect implementation processes. Such problems are also consistently shown to be centrally important in studies informed by psychological theories [9].

Future studies will reveal taxonomies of the contextual factors at work in implementation processes [10, 11]. However, we can think differently *now* about the place of context in implementation research. Contexts are dynamic: contextual factors that might constitute barriers to implementation in one place may facilitate it in others. Understanding how this happens will, in turn, help us to understand two key problems: how implementation processes may lead to ‘scaling up’ (changes in state over time, characterised by increasing the volume of activity in a social system) and how they may lead to ‘scaling out’ (changes in state across space, characterised by diffusion or spread between settings). They may also help us to both understand how some interventions fail to ‘stick’ in everyday practice settings and why it is so difficult to de-normalise already embedded practices. These are important problems in implementation research [12–14]. These are not just problems in trials. They extend across policy-led interventions too. Changes in the organisation of clinical work in surgery [15, 16], the delivery of telemedicine systems at scale [17, 18] and the scaling out and spread of new health technologies [19, 20], all provide evidence of the role of environmental mechanisms in shaping interventions.

### Purpose of this paper

Our aim in this paper is to develop and extend Normalisation Process Theory (NPT), to address the problem of context in implementation research. We locate the place of this paper in the development of NPT in Table 1, and define key terms in Table 2, showing how work to develop NPT began by asking what factors promote or inhibit the routine incorporation of complex interventions in everyday work [21, 22] and then moved on to develop a generic theory of implementation [23, 24]. More recently, NPT has engaged with the problem of dynamic elements of context as both the sources of resources that can be mobilised by participants in implementation processes [25] and as sources of turbulence that affect their course and direction [26]. At each stage in the development of NPT, we have been concerned with its utility as a way of understanding the dynamics of complex intervention trials [27–29]. In this paper, we extend NPT by engaging with ideas about complex adaptive systems. This will help us to better understand factors that affect implementation processes not just as part of highly controlled and structured complex intervention trials but also as larger scale implementation processes that sit outside of structured and protocol-driven projects. The paper has four objectives.

**Table 1:** Development of Normalisation Process Theory

Theoretical focus	Theoretical content	Research questions	Empirical focus
1 Users’ interactions with objects in implementation processes (2006)	Analysis of mechanisms of collective action (interactional workability, relational integration, skill set workability, contextual integration) [22, 23].	What factors promote or inhibit the routine incorporation of complex interventions in practice? How do they affect implementation processes and outcomes?	How complex interventions are operationalised by their users.
2 Agency within implementation processes (2009)	Analysis of mechanisms of agents’ contributions to implementation processes (sense-making, cognitive participation, collective action, reflexive monitoring) [24, 25].	What factors promote or inhibit the implementation, embedding and integration of practices? How do they affect implementation processes and outcomes?	The work people do when they implement a new technique, technology or organisational intervention.
3 Resource mobilisation in implementation processes (2013)	Analysis of social structural resources (roles, rules, norms and material resources) and social cognitive resources (potential and commitment) available to agents as they invest in implementation [26, 27].	What factors promote or inhibit the mobilisation of structural and cognitive resources for implementation? How do they affect implementation processes and outcomes?	How implementation processes work over time.
4 Implementation as adaptive self-organising in complex systems (this paper)	Analysis of properties of interventions as events in systems (plasticity and elasticity) and adaptive responses to emergence (normative and relational restructuring).	What factors promote or inhibit adaptation and self-organisation in complex systems? How do they affect implementation processes and outcomes?	How implementation processes differ between settings.

**Table 2:** Definition of concepts

Concept	Definition
Collective action	Participants in implementation contribute to their progress through work that achieves intervention coherence, cognitive participation, collective action and reflexive monitoring [23].
Context	Complex adaptive systems that form the dynamic environment(s) in which implementation processes are situated [25].
Coupling	Relations of dependence between actors, intervention components and dynamic elements of contexts.
Elasticity	The extent to which contexts can be stretched or compressed in ways that make space for intervention components and allow them to fit [89].
Emergence	The way in which the ‘global behaviour of a system results from the actions and interactions of agents’ [95] and unfolds unpredictably over time and across space.
Normative restructuring	Changes to the norms, rules and resources through which participation in implementation processes is structured.
Plasticity	The extent to which interventions and their components are malleable and can be moulded to fit their contexts.
Relational restructuring	Changes to the ways that participants in implementation processes are organised and relate to each other.

(i) To show how the contexts in which implementation processes are located can be characterised as complex adaptive social systems;
(ii) To characterise the importance of intervention plasticity (that is, the extent to which users can mould them to fit a particular context) and contextual elasticity (that is, the extent to which users can mould elements of the environment to allow a set of intervention components space to work);
(iii) To explain the ways that participants’ contributions to implementation processes (resource mobilisation and collective action) lead to adaptive work (normative and relational restructuring), and so shape implementation outcomes;
(iv) To characterise a set of verifiable propositions that can inform empirical investigations of healthcare interventions.

The paper is underpinned by a general theoretical argument, which is that *the generative mechanisms characterised by NPT are examples of self-organising mechanisms in complex adaptive social systems. Their operation explains differences in implementation processes over time and between settings, and they play an important part in determining intervention fidelity.*

## Implementation, context and complexity

Empirical research on implementation tends towards a quite pragmatic definition. It is constructed as *action in response to a call (or desire, or expectation, or command) for change* through which people are *asked (or want, or are expected, or are instructed) to do something new or different.* Normalisation Process Theory characterises a set of generative mechanisms of different kinds that motivate and give structure to the individual and collective action that stems from these calls.

Examples of these calls are abundant in healthcare, and they can be understood as initiating and sustaining the mobilisation of structural capacity (rules and resources), and cognitive potential (readiness and commitment), in the service of collective action. This collective action forms participants’ contributions to implementation processes [25]. As in our earlier work [22, 24], we define implementation as follows:

any deliberately initiated attempt to introduce new, or modify existing, patterns of action in health care or some other formal organisational setting. Deliberate initiation means that an intervention is: institutionally sanctioned; formally defined; consciously planned; and intended to lead to a changed outcome [22].

As we have pointed out [22, 23, 27], none of these takes place in a vacuum, and all of it is negotiated in diverse contexts. Indeed, context is the key problem here.

### The problem of context

Context presents implementation researchers with a problem because attempts to define and describe it have to encompass so much [30]. Starting with the UK Medical Research Council’s *Evaluation Framework for Complex Interventions* assertion of the many ‘moving parts’ in complex interventions [31, 32], structured attempts have been made to accommodate both complexity and emergence in context and to develop ways of including these in research design and evaluation models. Here, implementation framework developers building on Realist and Diffusion of Innovations Theories, such as Greenhalgh et al. [33], Rycroft-Malone et al. [34] and Damschroder et al. [35], have implicitly followed from Strauss et al’s. work on ‘negotiated orders’ [36–38]. In their influential *Consolidated Framework for Implementation Research*, Damschroder et al. [35] define inner and outer contexts, thus:

‘Generally, the outer setting includes the economic, political, and social context within which an organization resides, and the inner setting includes features of structural, political, and cultural contexts through which the implementation process will proceed (…) However, the line between inner and outer setting is not always clear and the interface is dynamic and sometimes precarious. The specific factors considered ‘in’ or ‘out’ will depend on the context of the implementation effort’ [35].

Defining context in this way makes clear the problem for the implementation researcher. Investigating and evaluating interventions and implementation processes in the setting of ‘whole systems’ is nearly impossible if these are understood in terms of diffuse factors related to entire economic and political systems, or the totality of structural and cultural forces that characterise the societies in which they are set. Of course, dynamic elements of context play a powerful role in shaping participants’ capacity and potential to respond to calls for practice implementation [25]. Even so, it is hard to accommodate the complicated and interdependent relationships between different structural elements of ‘whole systems’, or to track the pathways through which different macro-level actors and processes shape implementation contexts at meso- and micro-level [39–41].

Conceptualising context in terms of its relation to the individual—as in a concentric circle model, like that of Damschroder et al. [35]—raises questions about how to discriminate between factors that are at work far beyond the investigative remit of the researcher. Even when localised definitions of context are in play, this is a difficult task. McCormack et al. [42] make the underlying problem of scope abundantly clear.

The context in which health care practice occurs can be seen on one level as infinite as it takes place in a variety of settings, communities and cultures that are all influenced by (for example) economic, social, political, fiscal, historical and psychosocial factors. (…) the term context is used to refer to the environment or setting in which people receive health care services, or in the context of getting research evidence into practice, ‘the environment or setting in which the proposed change is to be implemented’ (…). In its most simplistic form, the term here means the physical environment in which practice takes place. Such an environment has boundaries and structures that together shape the environment for practice ([42] p96).

This perspective on context makes it into a *place.* It can be mapped to depict spatial relations between the loci of clinical practices, organisational systems and policy problems. Models that seek to link problems, activities and institutions in this way (e.g. Grembowski et al. [39]) are forceful reminders of the complicated nature of healthcare systems. Following on from the MRC framework, a review by Pfadenhauer et al. [30] has sought to build workable definitions of context.

Context is conceptualised as a set of characteristics and circumstances that consist of active and unique factors that surround the implementation. As such it is not a backdrop for implementation but interacts, influences, modifies and facilitates or constrains the intervention and its implementation. Context is usually considered in relation to an intervention or object, with which it actively interacts. A boundary between the concepts of context and setting is discernible: setting refers to the physical, specific location in which the intervention is put into practice. Context is much more versatile, embracing not only the setting but also roles, interactions and relationships [30].

Mapping context is useful, but understanding it as a *process* rather than a *place* may be more useful. It acknowledges that the context in which implementation takes place is the product of continuous accomplishments that require constant work to hold together and keep moving forward. There is a substantial body of research in organisational and economic sociology, going back 40 years, that has explored this insight [43–45]. In relation to this, Clark et al. [46] can describe the implementation of a stroke rehabilitation programme as,

a process involving the mobilisation of human, material, and organisational resources to change practice within settings that have pre-existing structures, historical patterns of relationships, and routinised ways of working [46].

However, a criticism from some clinical trialists has been that NPT focuses on agency at the expense of contextual mechanisms and constraints [47]. For example, Clarke et al. [46] assert that:

While May et al. (…) acknowledge that the NPT generative mechanisms are in dynamic interaction with local contexts and external drivers, the framework primarily addresses the mechanisms. Indeed, the theory tends to place undue emphasis on individual and collective agency without explicitly locating this within, and as shaped by, the organisational and relational context in which implementation occurs [46].

NPT studies (e.g. [18, 48–52]) have focused on *contextual* and *relational integration* [21, 23]—the work that actors do to when they realise and execute interventions in organisational and relational settings. What NPT does not do, however, is provide its own discrete theory of organisational structure and behaviour. There is no need for this. First, it can easily join with other theories that have this problem at their heart, even though these may be very different—for example, Strategic Action Field Theory [53]; Organisation Process Theory [43] and the ecological approach to theorising organisation taken by Allen [54–56] in her important ethnographies of hospital work. Second, as an implementation theory, NPT cannot assume that formal organisational settings are where the action is, or that professionals are the people doing it. Work by Kennedy et al. [57] and Gallagher et al. [58], for example, applies NPT to situations in the home and outside of formal healthcare organisations, where ‘contexts’ are not so much organisational as organising and are distributed across informal social networks [59, 60]. Implementation processes are sets of accomplishments, and their contexts are non-linear, emergent and dynamic [61, 62].

### Context is a dynamic accomplishment

Some of the most important insights into the complex and emergent relations between interventions and their contexts can be found in the work of Penelope Hawe and colleagues [1, 63, 64]. They have emphasised that,

Conventional thinking about preventive interventions focuses over simplistically on the “package” of activities (…). An alternative is to focus on the dynamic properties of the context into which the intervention is introduced. (…) An intervention may then be seen as a critical event in the history of a system, leading to the evolution of new structures of interaction and new shared meanings [63].

They go on to argue that,

“Complex” might be more appropriately ascribed to the system into which an intervention is introduced. Interventions might be best thought of as a time limited series of events, new activity settings and technologies that have the potential to transform the system because of their interaction with the context and the capability created from this interaction. (…) To make an intervention truly “an event” in the existing system, that is to meet the definition of an event being defined as “something *significant* that happens” then the intervention would need to change the future trajectory of the system’s dynamics [63].

What would it mean to change the ‘future trajectory’ of these dynamics? In this section of the paper, we explore the ways that ideas about complexity and emergence can help us think through the problem of context not as a fixed organisational structure or institutional entity but as an unstable, unfolding, process. It also helps us to consider the problem of process *evaluation* studies. As Oakley et al. [65] suggest, these focus attention on the behaviour of intervention participants, the activation and delivery of intervention components and the role of contextual factors in shaping intervention outcomes. Oakley et al. also draw attention to a crucial—but less often addressed—value in process evaluation in clinical trials, the capacity to distinguish between ‘interventions that are inherently faulty’ (where the intervention concept itself fails) and interventions that ‘are badly delivered’ (where implementation failure is at issue). The chief message of the growing body of ‘process evaluation’ studies of clinical trials is that context matters and that it is often the contextual dynamics of interventions that matter most. Process evaluations by Hooker et al. [66, 67], Kennedy et al. [51, 68], Bamford et al. [69], Clarke et al. [46] Godfrey et al. [70] and Ong et al. [71], all show how complex intervention trials are shaped in this way. For example, Bamford et al. point to the failure to take such factors into account as an important reason for the failure of the CAREDEM Trial [69] to achieve its objectives:

The primary focus during implementation was on the case managers as isolated individuals, with little attention being paid to the social or organizational context within which they worked. (…) Barriers relating to each of the four main constructs of Normalization Process Theory were identified: with a lack of clarity over the scope and boundaries of the intervention (coherence); variable investment in the intervention (cognitive participation); a lack of resources; skills and training to deliver case management (collective action); and limited reflection and feedback on the case manager role (reflexive monitoring) [69].

Thought about as a set of ongoing accomplishments, rather than as concrete structures, the way we understand ‘contexts’ might change. Complex Adaptive Systems Theory is useful here, because it focuses attention on fundamental mechanisms of emergence. This perspective has played an important part in debates about healthcare organisation and delivery, like those set in train by Plsek and Greenhalgh [72]. It has proved attractive to clinicians, managers and health policy-makers because of assumptions about the dynamic, variable and unpredictable behaviours of interventions and their environments.

Complex Adaptive Systems Theory remains relevant in thinking about healthcare processes because it provides a way of ‘embracing [the] intrinsic system uncertainty’ ([73] p. 70) that seems to be one of their most important characteristics in practice. The notion that complex systems are ‘self-organising’ runs through this literature and suggests a tendency to return to equilibrium states:

dynamic and resilient systems that have the capacity for self-organisation and self-stabilising adaptation in the face of turbulent, literally chaotic, challenges from the environmental supra-systems in which they are embedded. When the level of chaos in the environment is so great as to threaten the integrity of these complex systems, the systems are able to transform themselves (self-organise) into higher order levels of organisation, with increased structural complexity and seemingly enhanced coherence, and with internal, yet still dynamic, stability leading to a new equilibrium ([74] p. 616).

Complex adaptive systems theorists propose that ‘simple rules’ govern the unfolding of complex systems [75]. These offer a means of understanding the emergent behaviour of healthcare organisations, professionals and patients [76–79]. However, much less attention has been paid to the *adaptive* mechanisms at work in complex adaptive systems. Social systems are self-organising and self-stabilising only to the extent that human actors invest effort in making them so and only to the degree that human actors work with mechanisms that produce and sustain self-organisation. Gunn et al. [80] point to the relevance of such a view to a study of the implementation of a new model of care for people with depression:

To explore the context of primary care and the way it responds to people experiencing depression, our approach was informed by the view that primary care is a complex adaptive system (CAS) (…). [These] consist of different members and components that are dynamic, interactive, and dependent. These systems are adaptive with the capacity to change and to self-organise; they have shadow systems operating in daily work; they have emergent properties that are more than the sum of individual parts, and show initial conditions that can markedly influence what happens in practice [80].

The questions that arise from this are about how adaptive mechanisms can be characterised and how they work. It is to these that we turn next. In this section of the paper, we have argued that NPT characterises generative mechanisms that drive implementation processes and that these processes take place in contexts that can be characterised as complex adaptive systems. Their participants’ actions shape, and are shaped by, the mechanisms at work in these systems.

### Coupling: tight and loose

There are two important consequences of understanding the contexts in which implementation processes are enacted in terms of complex adaptive systems. First, it acknowledges that these processes are emergent. They unfold, over time and between settings, and are shaped by many different factors. Interactions between these factors may lead to turbulence and other unanticipated effects. Second, it means that we ought to consider the work of implementation not just in terms of operationalising some new technique, technology or organisational practice but also in terms of accomplishing order and predictability and damping down turbulence. Against this background, the mechanisms that are characterised by NPT also define the organisational work of shaping emergence and holding back unanticipated consequences.

Collective action, rather than individual behaviour, is at the centre of this work because complex intervention trials—like many other kinds of implementation problem—so often involve one group of participants (the trial team) intervening in a context that they do not control by calling on another group of participants to change their work or to do new work. What matters here is how intervention components are coupled to each other and how they are coupled to dynamic elements of context [1, 25, 63]. The more tightly coupled intervention components are, the less discretion in resource mobilisation and actors’ contributions are available to participants in their implementation and the less traction the intervention gains.

The more loosely coupled intervention components are, the more discretion in resource mobilisation and actors’ contributions are available to participants in their implementation. Taft et al. [66, 67, 81, 82] describe the MOVE trial, a complex intervention study with a bundle of intervention components aimed at identifying and supporting women at risk of, or experiencing, family violence (FV). There were multiple intervention components. At the centre of the trial was a screening instrument that included questions intended to facilitate detection of family violence. This was offered to all women seen by eight teams of Maternal and Child Health Specialist Nurses in Victoria, Australia. Sitting behind this was a clinical practice guideline and a clinical pathway that mapped possible courses of action available to nurses if risk or experience of FV was detected. Finally, specialist FV liaison workers offered support for Nurses. However, while there were many intervention *objects*, the mission critical element of the trial was the creation of an interactional space in which women felt safe to disclose FV and the availability of a structured script for action that nurses felt safe to use. A transaction space was thus created in which social structural and social cognitive resources could be mobilised [25] and in which the intervention was workable and integrated in its operational context [21]. Women had discretion about whether or how to disclose FV. The context was characterised by multiple layers of operational complexity and emotional turbulence. With intervention components coupled in this way, the negotiation space in which they were to be enacted was known to nurses delivering the intervention. This trial was successful. It had positive outcomes [82]. More than this is was implemented and sustained. Two-year follow-up [67] showed that the nurses had decided to continue with this work after the end of the trial and had normalised the intervention components into their practice.

### Contexts are negotiated

To understand organisational aspects of implementation better, we need to explore the ways that they are shaped by the behaviours and actions of participants as they negotiate the normative and relational environment in which they are set. These negotiations mediate between implementation (resource mobilisation and actors’ contributions) and their outcomes (experienced workability and integration [21] and the embeddedness of interventions [23]). An important consequence of these negotiations is that they lead to different kinds of restructuring: we can define two of these.

N*ormative restructuring* occurs when negotiating the implementation of intervention components in a complex adaptive system leads to modifications to the conventions, rules and resources that participants experience as providing the scaffolding for everyday behaviour and action. Here, ‘successful’ interventions seem to ‘restructure and reinforce new practice norms and associate them with peer and reference group behaviours’ ([12]: p. 12). Normative restructuring leads to changes in participant behaviour and system dynamics over time, but it also involves interactions with intervention components themselves. An important property of those components is their *plasticity*—the extent to which their users experience them as malleable and can mould them to fit their immediate contexts. The more plastic intervention components are, the more that their users have discretion about how to deploy them in practice.

R*elational restructuring* occurs when negotiating the implementation of intervention components in a complex adaptive system leads to changes in the structure and conduct of the interpersonal interactions and group processes that make collective action possible. As participants enact their contributions to an implementation process, their accountabilities to each other are reworked. Here, an important property of the expression of those relations is their *elasticity*—the degree to which they can be stretched and moulded to give users room for manoeuvre as they operationalise intervention components.

The degree of user discretion that stems from the experienced plasticity of intervention components and the room for manoeuvre that users find when implementation environments are characterised by elasticity are important. When intervention components are inflexible and rigidly applied, they require high levels of commitment from their users—and where this cannot be guaranteed, they require specialist practitioners or facilitators—because the turbulent flows and varying magnitude of events that are associated with complex adaptive social systems make them difficult to routinely embed in practice. Inelastic implementation environments are often characterised by rigidly formed group processes and inflexible and impermeable organisational structures. These reduce the room for manoeuvre available to participants in implementation processes and mean that the transportability of intervention components between settings is inhibited.

In the WISE Trial in England, for example, Kennedy et al. [51, 68, 83] showed how attempts to implement a self-management intervention for long-term conditions in a primary care setting failed because of the relationship between the degree of plasticity possessed by a set of intervention components and the degree of elasticity possessed by the normative and relational structure of host contexts. The WISE intervention was *plastic* and assumed that participants possessed a high level of autonomy over their engagement with the intervention and assigned to them a high degree of discretion about how they delivered it. However, the intervention was enacted in an inelastic context of practice (including the technical division of labour and the poor integration of the mix of capitation and fee for service funding models in English Primary Care). Restructuring—normative or relational—was simply not possible. Kennedy et al. [57, 84–86] overcame this by shifting the focus of their interventions to the normative and relational restructuring of *informal* social networks, where intervention components and implementation participants were much more loosely coupled. This led to a successful intervention.

Questions about restructuring and the properties of interventions and implementation environments reflect longstanding debates about the place of ‘fit’ in conceptual models of implementation [87–90]. There is, however, something much more fundamental going on here. Participants in implementation processes need to work to sustain an orderly pattern of social interactions and relations and a predictable flow of events in the face of complexity. This work is a basic underpinning of all forms of human association [91, 92]. So, normative and relational restructuring can be observed as continuously taking place over time and between settings. When intervention studies ‘fail’, it may be because participants have been unable to perform the degree of restructuring that is necessary to do implementation work. Restructuring is an important, but poorly understood, *adaptive* element of implementation processes.

### Conceptualising the relationship between NPT and the context of action

**Fig. 1:**
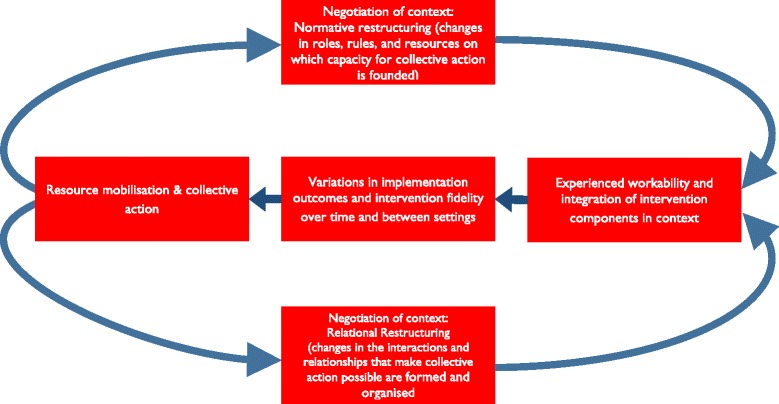
Implementation is a set of feedback loops, not a linear process

The focus of this paper has been on how we should understand the dynamics of implementation processes in relation to their contexts. An important message of the paper is that contexts are dynamic and are subject to restructuring processes that take within complex adaptive social systems. We present this graphically in two ways. In Table 1, we show how NPT has developed over time to uncover the elements of implementation processes described in this paper. In Fig. 1, we show how these elements relate to each other and define a process made up of feedback loops that continuously shape and reshape implementation fidelity and outcomes. This occurs not just over time but also between settings as contextual factors affect implementation processes. The theoretical assumptions about the *adaptive* work that this involves lead us to six propositions about implementation processes and about the sources of variations in their outcomes. First, we propose that variations in intervention fidelity and outcomes are the products of observable mechanisms.

**Proposition 1a**. *Differences in participants’ resource mobilisation and actors’ contributions explain variations in process and negotiation outcomes over time.*

**Proposition 1b**. *Differences in normative and relational restructuring processes explain variations in process and negotiation outcomes between settings.*

In turn, these lead to further propositions about the role of intervention components and practice contexts in ensuring workability and integration in practice. They also suggest important sources of problems in fidelity to interventions. We propose that the relative plasticity of interventions and the elasticity of contexts have important consequences for implementation processes. This leads to a second pair of propositions about the properties of intervention components and the contexts in which they are enacted.

**Proposition 2a**. *The greater the degree of plasticity possessed by a set of intervention components, the less strain that actors enacting them place on the normative and relational structure of host contexts.*

**Proposition 2b**. *The greater the degree of elasticity possessed by the normative and relational structure of host contexts, the less strain they place on actors enacting a set of intervention components.*

Taken together, these propositions help us to explain why intricately and rigidly structured complex intervention trials often lead to negative outcomes and why an important subset of implementation processes ‘fail’. We propose that the degree of coupling of intervention components and the degree of discretion available to actors are critical to outcomes.

**Proposition 3a**. *The more tightly coupled intervention components are, the less discretion participants have in resource mobilisation and collective action, and the more they must do adaptive work to ensure intervention integration.*

**Proposition 3b**. *The more loosely coupled intervention components are, the more discretion participants have in resource mobilisation and collective action, and the more they must do adaptive work to ensure intervention workability.*

Here, the tight coupling of intervention components and limits on discretion about action leads to the minimisation of available space for discretion and negotiation. These propositions—and the theory that underpins them—help us to understand how participants’ agentic contributions to implementation processes, which is the main focus of NPT, interact with and are shaped by the contexts in which action takes place.

## Conclusions

Debates about the relationships between implementation processes and their contexts replicate in applied settings, important, longstanding and fundamental problems in sociology, psychology and economics. In those disciplines, the central problem is the relationship between social and economic structures and individual and collective agency [93, 94]. These higher order debates remain unresolved and are likely to remain so for the foreseeable future. Even so, all theories, frameworks and models of implementation—irrespective of their disciplinary orientation—offer ways to reflect on the relationship between the things that individuals and groups do and the social contexts in which these actions are embedded. This suggests that implementation research is an important laboratory for investigating actors’ contributions and dynamic features of context that shape self-organisation in complex adaptive social systems. These problems lead to fundamental questions in the social sciences. Indeed, the search for good theories of implementation represents just one of these fundamental questions which is how can we best understand the dynamics of human agency under conditions of constraint?

Our aim in this paper has been to advance understanding of interactions between implementation activities and their contexts. We have suggested that the implementation mechanisms specified by NPT are relevant to *adaptive self-organisation* in complex and emergent social systems. These mechanisms are important elements of implementation processes. One conclusion of our development of NPT in this paper is that the outcome of implementation processes may depend on the plasticity of intervention components; the degree of participants’ discretion over resource mobilisation and actors’ contributions; the elasticity of contexts and the extent of normative and relational restructuring. These consequences of the negotiation of intervention components in practice have important implications for the design and conduct of randomised controlled clinical trials of complex healthcare interventions. They also help us understand why such methods may not be best suited for evaluating complex interventions in dynamic environments.

Future work will explore the methodological implications of NPT suggested by this paper. In particular, it will respond to the problems of study design, measurement and evaluation raised by understanding implementation processes as non-linear, emergent and dynamic events within systems. In theory development, we will explore ways to conceptualise the interactions between the micro- and meso-level theoretical propositions on which NPT has been built and macro-level models through which we can explore the workings of ‘whole systems’ and their effects.

The ecological perspective offered by Normalisation Process Theory has implications for understanding broader processes of socio-technical change. It raises questions about the common definition of implementation processes as clearly defined, linear, finite projects. Scaling up and scaling out are the translational efforts that take healthcare interventions beyond the closed system of the evaluation study into ‘real world’ contexts. Understanding these processes in terms of feedback loops, adaptation mechanisms and the normative and relational compromises that stem from them enables us to see the generative mechanisms of NPT as core elements of self-organisation in complex systems and to understand implementation processes as non-linear, emergent and dynamic.

## Abbreviations

CAREDEM: Collaborative cARE for people with DEMentia in primary care (Trial Acronym)
CAS: Complex adaptive system
FV: Family violence
MOVE: Improving maternal and child health nurse care for vulnerable mothers (Trial Acronym)
NPT: Normalisation Process Theory
TRACS: Randomized Controlled Trial of Caregiver Training after Stroke (Trial Acronym)
WISE: Whole System Informing Self-management Engagement (Trial Acronym)
